# Navigating the outbreak: a comprehensive analysis of pediatric *Mycoplasma pneumoniae* pneumonia via targeted next-generation sequencing in Wuhan, 2022–2023

**DOI:** 10.1128/spectrum.02463-24

**Published:** 2025-04-08

**Authors:** Changzhen Li, Jingjing Rao, Xiaomei Wang, Lifang Feng, Yun Xiang, Feng Tang

**Affiliations:** 1 Department of Laboratory Medicine, Wuhan Children’s Hospital (Wuhan Maternal and Child Healthcare Hospital), Tongji Medical College, Huazhong University of Science & Technology, Wuhan, China; 2 Department of Endocrinology, Wuhan Children’s Hospital (Wuhan Maternal and Child Healthcare Hospital), Tongji Medical College, Huazhong University of Science & Technology, Wuhan, China; The George Washington University School of Medicine and Health Sciences, Washington, DC, USA

**Keywords:** targeted next-generation sequencing, *Mycoplasma pneumoniae *pneumonia, non-*Mycoplasma pneumoniae *pneumonia, serum amyloid A, lymphocyte subsets

## Abstract

This study aims to delineate the epidemiological characteristics and impacts of the *Mycoplasma pneumoniae* (MP) outbreak, utilizing targeted next-generation sequencing (tNGS) to assess pathogen prevalence and co-infections in pediatric pneumonia cases. Pediatric patients admitted to Wuhan Children’s Hospital with pneumonia from 1 October 2022 to 31 October 2023 were included. tNGS was used for comprehensive pathogen detection, including MP and other respiratory pathogens, with additional sequencing of the 23S rRNA gene V region to identify macrolide resistance mutations. This study enrolled 10,223 patients with pneumonia with a positivity rate of 98.4% by targeted next-generation sequencing. Fever (86.4%) and cough (79.3%) were the most common symptoms of *Mycoplasma pneumoniae* pneumonia (MPP). Lung consolidation (25.8%) was a common imaging feature, and corticosteroid use was noted in 22.5% of MPP patients. MP proved to be the primary pathogen, particularly evident during the MP pandemic, which began in March 2023 and peaked in October with a detection rate of 63.2%. Of the 4,397 MPP cases, 34.5% were sole infections, while 65.6% were co-infections, mostly with viruses. The main causative agents of co-infections were *Haemophilus influenzae* and *Rhinovirus*. The macrolide resistance rate was 79.03%. The A2063G mutation in the 23S rRNA V region is the dominant mutation. High-sensitivity C-reactive protein and serum amyloid A were significantly elevated in MPP, while the absolute counts of CD3^+^T, CD4^+^T, CD8^+^T, CD19^+^B, and NK cells were significantly reduced. This study demonstrates the utility of tNGS in identifying MP co-infections and macrolide resistance and highlights the role of inflammatory markers and lymphocyte subpopulations in differentiating MPP from non-*Mycoplasma pneumoniae* pneumonia for clinical management.

**IMPORTANCE:**

Our study is of great scientific value as it provides practical solutions to clinical challenges and supports both clinical decision-making and public health policy. First, it presents new and important insights into the application of targeted next-generation sequencing (tNGS) technology, which enables rapid and accurate pathogen detection and overcomes the limitations of conventional diagnostic methods. Second, the large sample size, focusing specifically on children during a *Mycoplasma pneumoniae* epidemic, provides valuable epidemiologic data specifically for the Wuhan region. Finally, by integrating rapid tNGS detection with inflammatory markers and lymphocyte subsets, our study demonstrates direct clinical applications that have the potential to improve patient outcomes. This approach highlights the practical utility of our research in enhancing clinical decision-making and contributes important knowledge to the field.

## INTRODUCTION

*Mycoplasma pneumoniae (*MP) is one of the most important pathogens causing community-acquired pneumonia (CAP) in the pediatric population and accounts for about 30%–40% of diagnosed cases (1, 2). MP infections occur sporadically worldwide, with regional outbreaks occurring every 3–7 years, lasting 1–2 years each time (3), but the mechanism for this epidemic cycle remains unclear. Co-infection of MP with other respiratory pathogens is common, but it is not known with certainty whether co-infection is related to disease severity, and possible regional trends for co-infection in China remain unknown (4).

The lack of an organized surveillance program makes it difficult to assess the actual impact of MP on public health in China. The “Guidelines for the Diagnosis and Treatment of *M. pneumoniae* Pneumonia in Children (2023 edition)” emphasize that *M. pneumoniae* is the leading cause of community-acquired pneumonia in children aged 5 years and older in China. The MP pandemic has highlighted the importance of reliable surveillance and led to advances in pathogen detection technologies. One of these technologies is targeted next-generation sequencing (tNGS), which uses probe hybridization. This technology uses probe hybridization and capture or ultramultiplex PCR amplification to enrich nucleic acid sequences of a broad spectrum of pathogenic microorganisms. tNGS provides comprehensive coverage of common pathogens associated with respiratory infections with high sensitivity and specificity, making it a suitable tool for the surveillance of a wide range of pathogens, especially for multiple and difficult-to-cultivate pathogens (5).

Macrolides are the preferred treatment for MP infections. However, genetic mutations in domain V of the 23S rRNA of MP, such as A2063G, A2064G, A2067G, and A2617G, have been identified as factors contributing to macrolide resistance (6). Monitoring the prevalence and types of mutations in macrolide-resistant *Mycoplasma pneumoniae* (MRMP) is crucial for clinical diagnosis and treatment planning. MP invades airway epithelial cells by binding its P1 protein to cilia, triggering the secretion of pro-inflammatory cytokines in the airway mucosa. This process triggers an acellular inflammatory response that damages the tissue and impairs the host’s immune functions (7, 8). In addition, changes in T cell subsets are closely associated with the progression of MP infection. By activating polyclonal lymphocytes, MP disrupts the balance of T lymphocyte subsets, weakening the immune system and impairing the function of other immune cells (9). This imbalance is particularly important for the pathogenesis of MP infection in infants and young children (10). Therefore, the determination of serum inflammatory markers and lymphocyte subpopulations in children with MPP is crucial for determining clinical treatment strategies. Recent research has focused mainly on cytokines, lymphocyte subsets, and their role in refractory *Mycoplasma pneumoniae* pneumonia in children (11, 12) and on expression differences between normal and severe MPP cases (13). However, there are few studies on inflammatory and immune responses to sole or co-infection with MP in children.

The present study aims to fill this gap by analyzing the expression of relevant inflammatory proteins and immune markers in different infection modalities and thereby proposing new strategies for the differential diagnosis of MPP. In addition, this study also aims to determine the prevalence of MP in children with CAP in Wuhan, China, since 2022 using tNGS. It focuses on both MRMP and macrolide-susceptible *Mycoplasma pneumoniae* as well as co-infections with other respiratory pathogens.

## MATERIALS AND METHODS

### Study population and data collection

In our hospital, children diagnosed with pneumonia by the doctor usually undergo further tests to identify the causative pathogens. After obtaining signed informed consent from parents or guardians, clinical samples, including throat swabs (TS), sputum, or bronchoalveolar lavage fluid (BALF), were collected and analyzed with tNGS. The hospital began offering tNGS for respiratory samples in October 2022. In the initial months, the number of participants was limited as clinicians were unfamiliar with the sample collection process and ongoing adjustments to hospital policies for tNGS implementation. These initial challenges included staff training and establishing standard operating procedures for sample handling. As these issues were resolved and awareness increased, the number of cases enrolled increased significantly over the following months, reflecting the integration of tNGS into routine clinical diagnostics.

The aim of this retrospective observational study was to investigate infections caused by respiratory pathogens in children diagnosed with pneumonia, predominantly MPP, between October 2022 and October 2023. The inclusion criteria were as follows: (i) age younger than 18 years; (ii) hospitalization at Wuhan Children’s Hospital; (iii) radiologically confirmed pneumonia with at least one symptom or clinical sign suggestive of pneumonia, such as fever, cough, wheezing, dyspnea, or abnormal auscultation findings (14); (iv) performing tNGS of TS, sputum, or BALF specimens available for standard procedures ≤48 hours after admission; (v) the diagnosis of MPP was based on a combination of clinical symptoms, radiologic findings, and laboratory results, including molecular methods, according to the *Guideline for diagnosis and treatment of community-acquired pneumonia in Children* (14,15) and the *Diagnosis and Treatment Guidelines for Mycoplasma Pneumonia in Children* (15); and (vi) a signed informed consent form from the legal guardians of all participants that allowed for sample collection and tNGS testing. The control group consisted of healthy children who underwent routine health examinations at our hospital during the study period. The inclusion criteria for the control group were (i) age under 18 years, which corresponds to the age range of the study population; (ii) no history or clinical signs of respiratory tract infections, systemic inflammatory conditions, or significant illnesses within the 4 weeks prior to sampling; (iii) no chronic conditions, such as asthma, cystic fibrosis, or primary immunodeficiency. Data collected for this analysis included demographic information, respiratory specimen sources, pathogen identification, and laboratory test results, with coinfection defined as the detection of multiple positive pathogens in the same sample.

### tNGS respiratory pathogen 100 plus assay

The TS, sputum, and BALF samples were sent to a commercial company laboratory (Respiratory Pathogen 100 Plus Panel, KingMed Diagnostics, Guangzhou, China) for tNGS analysis for respiratory pathogens and drug resistance genes. An early diagnosis of respiratory tract infections was made by detecting 153 pathogens, including 65 bacteria, 68 viruses (25 DNA viruses and 43 RNA viruses), 14 fungi, and 6 mycoplasma/chlamydia, as well as 3 types of antimicrobial-resistant organisms with 15 genes (carbapenem-resistant *Enterobacterales*, methicillin-resistant *Staphylococcus aureus,* and macrolide-resistant MP at four mutation points of the 23S rRNA at A2063G, A2064G, A2067G, and C2617G) according to the manufacturer’s instructions (16, 17). After nucleic acid extraction, specific primers were designed to amplify target sequences of bacteria, mycobacteria, viruses, fungi, and selected antimicrobial resistance genes and enrich these multiplex targets for library preparation and subsequent NGS sequencing (18, 19).

Sequence reads (normalized to 100,000 starting sequences) were calculated using the internal controls provided in the report. Exogenous plasmids with known concentrations were used as internal controls to estimate the pathogen concentration. The normalized read count of the target pathogen was then derived from the amplification efficiency relative to the internal control. To increase the precision of pathogen identification, we systematically excluded organisms that are typically part of the normal oral microbiota from our analysis, including *viridans streptococci*, *Streptococcus anginosus* group (consisting of *Streptococcus anginosus*, *Streptococcus intermedius*, and *Streptococcus constellatus*), *Neisseria mucosa*, *Corynebacterium* species, *Prevotella* species, and *Fusobacterium nucleatum* (20). Although Epstein-Barr virus (EBV) and *Candida albicans* are commonly considered commensals in healthy humans, previous studies have reported their potential role as pathogens in respiratory infections (21–23). Based on these findings, they were included in the analysis of our study as potential pathogens.

### Inflammatory proteins and flow cytometric assay

The Siemens BN II system (Germany) was used for the detection of high-sensitivity C-reactive protein (hsCRP) (24), and the concentration of the proinflammatory marker serum amyloid A (SAA) was measured in plasma samples using the AFS2000A dry fluorescence immunoassay analyzer (Weimi Technology, Guangzhou, China) according to the manufacturer’s instructions. The absolute counts and percentages of lymphocyte subpopulations (CD3^+^T, CD4^+^T, CD8^+^T, CD19^+^B, and NK cells) were determined using flow cytometric bead assay kits (BD Company, USA) and analyzed with the FACSCANTO II flow cytometer (BD Company, USA) (25). For the flow cytometric analysis, we used a multi-gating approach in which fluorescently labeled antibodies are used to identify important cell surface markers, such as CD4 and CD8. Specifically, two reagent panels were used: (i) CD3-FITC, CD8-PE, CD45-Percp, and CD4-APC; and (ii) CD3-FITC, CD16/CD56-PE, CD45-Percp, and CD19-APC. In addition, forward scatter and side scatter measurements were performed to assess cell size and granularity and ensure accurate identification of lymphocyte populations. To minimize the presence of dead cells that could potentially interfere with analysis, venous whole blood samples were transported to our laboratory within 2 hours of collection, preserving the integrity of the samples for accurate immunoprofiling. Since hsCRP and SAA were not tested in the healthy control group of children, we set the normal values for hsCRP at 0–3 mg/L and for SAA at 0–10 mg/L based on recommendations from instrument and reagent manufacturers and literature reports (26, 27).

### Statistical analysis

SPSS software (version 19) was used for statistical analysis. The normal distribution data were expressed as medians and interquartile ranges (IQRs). Data were tested for normality using the Shapiro-Wilk test, and Mann-Whitney *U* tests were used to directly assess pairwise group differences without the need for additional *post hoc* procedures. Categorical variables were analyzed using the chi-square test or Fisher’s exact test. Multivariate regression models for length of hospital stay and clinical management data were used to account for potential confounders, including demographic variables such as geographic distribution and underlying diseases. In univariate analyses with multiple comparisons, the Benjamini-Hochberg correction was used to adjust *P*-values and control for false discovery rate. Effect sizes are reported for Mann-Whitney *U* (*r*) and chi-square tests (Cramér’s V), both interpreted in the same way: small effects  = 0.1, medium effects  =  0.3, and large effects  =  0.5 (28, 29). A *P*-value of less than 0.05 was considered statistically significant.

## RESULTS

### Prevalence of pediatric *Mycoplasma pneumonia*

**Fig 1:**
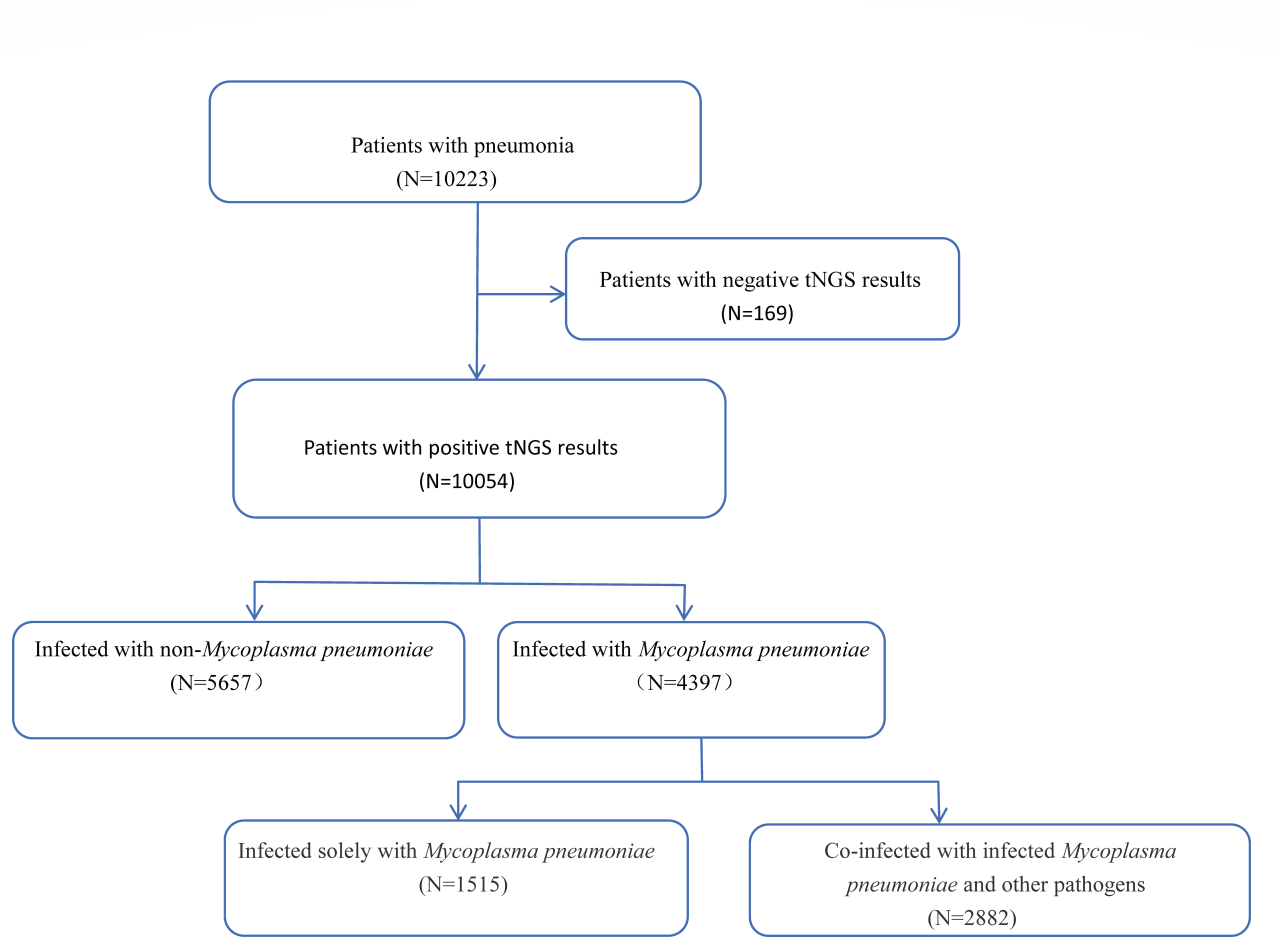
Flowchart illustrating the methodology for identifying and analyzing MPP cases in pediatric patients.

In this study, respiratory samples from 10,223 pediatric patients with pneumonia between October 2022 and October 2023 were analyzed using preliminary tNGS. After excluding 169 patients (1.7%) with negative tNGS results, the final cohort comprised 10,054 patients (98.4%). Of these, 4,397 patients (43.0%) were identified as infected with MP, including 1,515 patients with sole MP infections and 2,882 patients with co-infections with MP and other pathogens. The remaining 5,657 patients had infections that were not associated with MP (Fig. 1).

**Fig 2:**
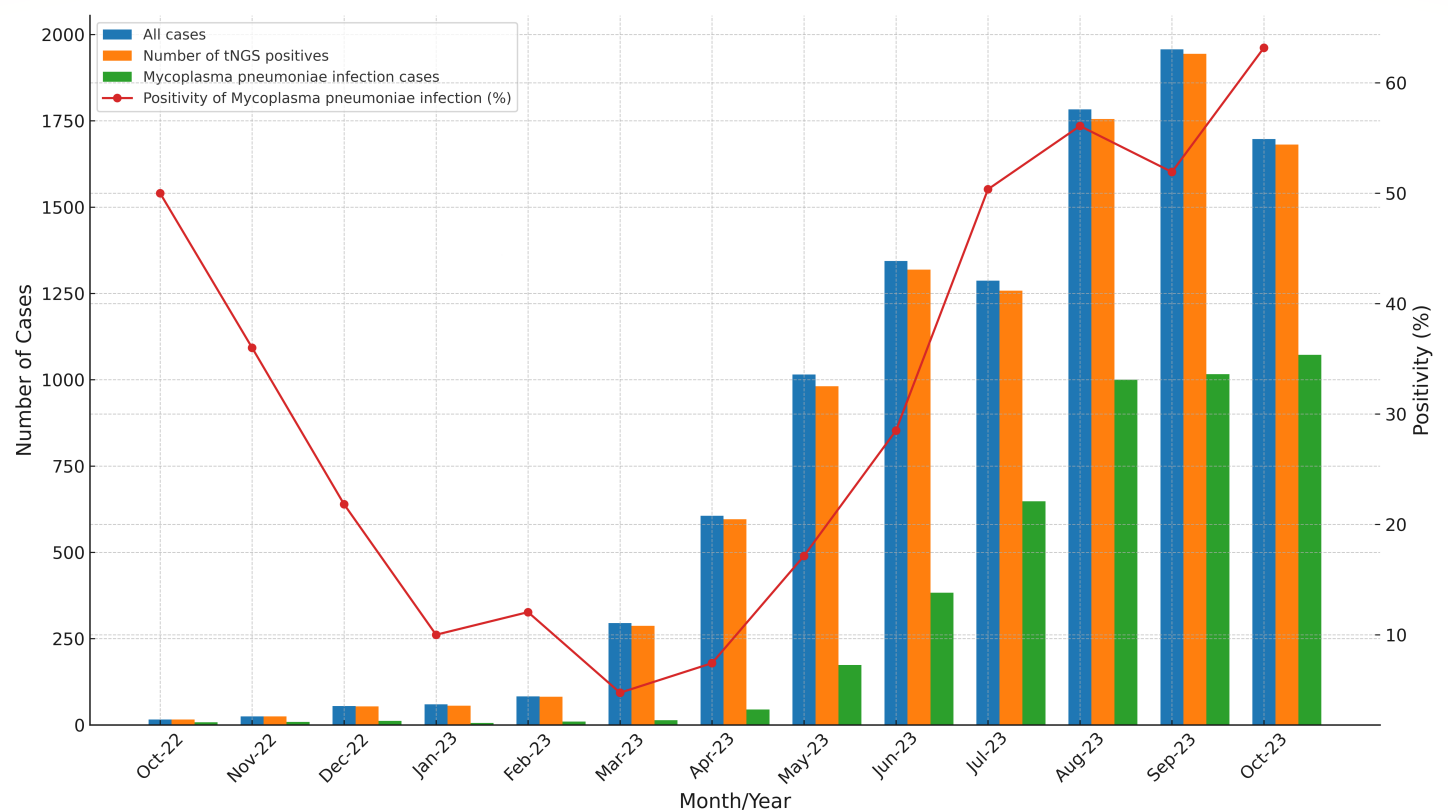
Monthly trend of pediatric pneumonia cases, tNGS positivity, and MP infection rates from October 2022 to October 2023.

Our results show that a positive pathogen was detected in over 98% of the samples collected each month in the entire pediatric pneumonia cohort. Despite this stability, there was notable variability in positivity rates of MP infection. From October 2022 to February 2023, a declining trend was observed, with the detection rate dropping from 50% to a low of 12.5%. However, from March 2023, there was a significant rebound in detection rates, which increased steadily each month, reaching a peak of 63.2% in October (Fig. 2).

**Fig 3:**
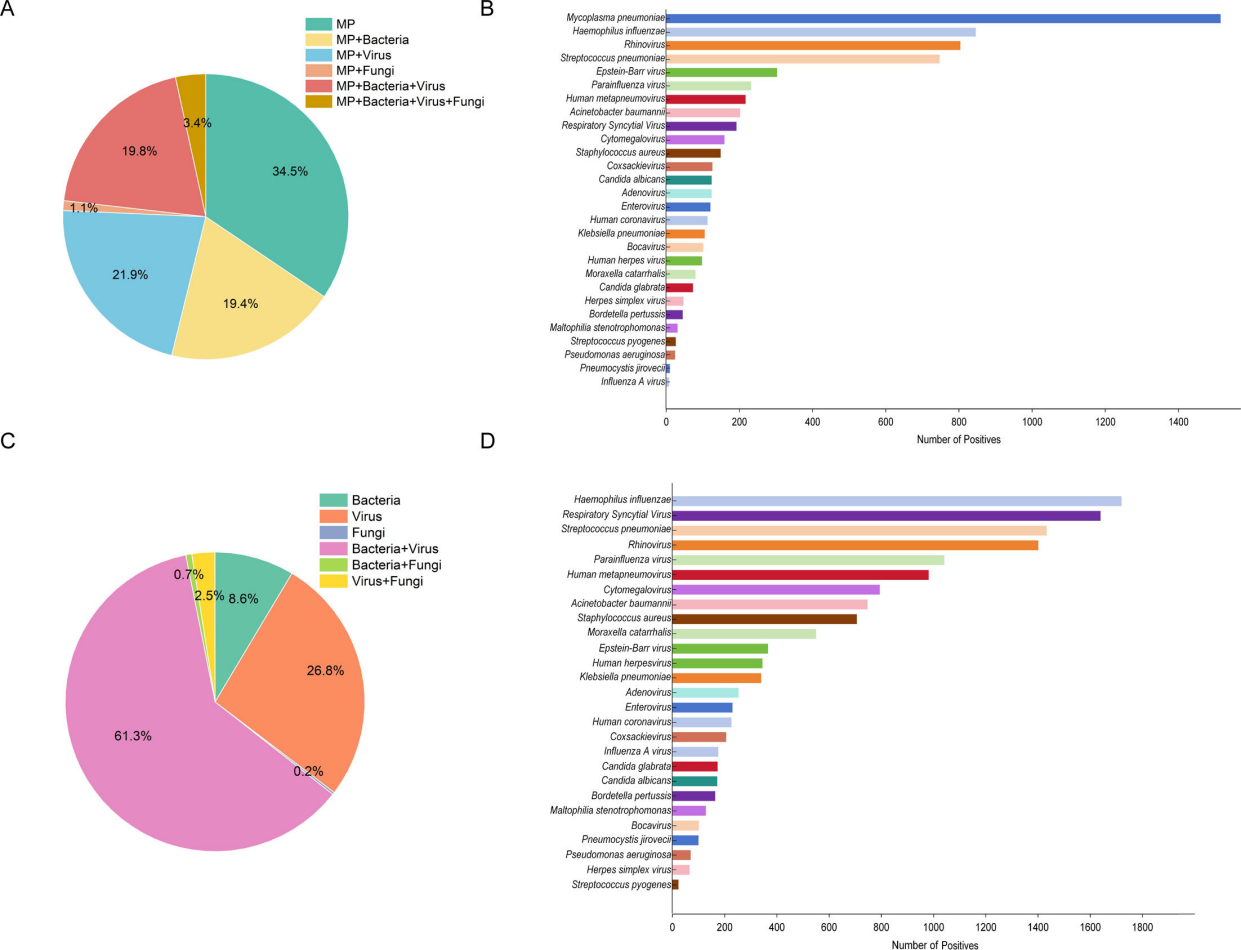
(**A**) Pie chart showing the proportions of different pathogen types (bacteria, viruses, and fungi) identified in pediatric patients with MPP. (**B**) Bar chart showing the number of various pathogens identified in pediatric patients with MPP. The chart provides a detailed breakdown of pathogen types co-infected with MP and highlights the distribution of bacterial, viral, and fungal pathogens. (**C**) Pie chart showing the proportions of various pathogen types (bacteria, viruses, and fungi) identified in pediatric patients with NMPP. (**D**) Bar chart illustrating the number of different pathogens identified in pediatric patients with NMPP.

Of the 4,397 MPP cases, 34.5% were sole infections and 65.5% were co-infections. In co-infections, MP was detected together with bacteria in 19.4% of cases, with viruses in 21.9% of cases, and with a combination of bacteria, viruses, and fungi in 3.4% of cases (Fig. 3A). The number of specific pathogens also illustrated the diversity of microbial infections in the cohort. In addition to MP, *Haemophilus influenzae*, *rhinovirus,* and *Streptococcus pneumoniae* were detected at high rates, and other notable pathogens included Epstein-Barr virus and parainfluenza virus (Fig. 3B). Among the 5,657 patients infected with non-*Mycoplasma pneumoniae* pneumonia (NMPP), bacterial and viral co-infections were the most common type of infection at 58.3%, while bacterial infections were found exclusively in 25.5% of patients and viral infections exclusively in 8.2% (Fig. 3C). Specific pathogen analysis showed that *Haemophilus influenzae* was the most frequently detected pathogen, followed by *respiratory syncytial virus*, *Streptococcus pneumoniae*, *rhinovirus,* and *parainfluenza virus*. In addition, *human metapneumoviruses*, *cytomegaloviruses,* and *Acinetobacter baumannii* were also detected at high rates (Fig. 3D).

### Symptoms, management, and imaging characteristics

Among the 4,397 pediatric patients with MPP, fever (86.4%) and cough (79.3%) were the most frequently reported symptoms, both significantly higher than in NMPP patients (66.3% and 57.1%, respectively, Cramér’s V = 0.231 for fever, Cramér’s V = 0.233 for cough, *P* < 0.001). Wheezing was more frequently observed in NMPP patients and those with co-infection involving MPP, compared to sole MPP patients.

Symptoms such as vomiting and diarrhea were rare in both groups. Within this cohort of pediatric MPP patients, 166 (3.8%) had underlying systemic diseases. Recurrent respiratory infections were the most common (50 cases, 1.1%), followed by malnutrition (34 cases, 0.8%), congenital heart defects (25 cases, 0.6%), bronchopulmonary dysplasia (27 cases, 0.6%), neurological disorders (17 cases, 0.4%), malignant tumors (9 cases, 0.2%), nephrotic syndrome (7 cases, 0.2%), immunodeficiency disorders (7 cases, 0.2%), and asthma (6 cases, 0.1%). Regarding clinical management, admission to the ICU was less frequent in MPP patients (1.5%) than in NMPP patients (3.7%, Cramér’s V = 0.065, *P* < 0.001). Oxygen therapy was required less frequently in MPP patients (4.5%) than in NMPP patients (12.5%, Cramér’s V = 0.139, *P* < 0.001). Ventilatory support was rare in both MPP and NMPP groups, with a significantly higher proportion of NMPP patients (1.9%) requiring ventilatory support compared to MPP patients (0.5%) (Cramér’s V = 0.063, *P* < 0.001). However, the use of corticosteroids was significantly higher in MPP patients (22.5%) than in NMPP patients (11.3%, Cramér’s V = 0.251, *P* < 0.001). Imaging features in MPP patients showed a higher frequency of consolidation (25.8% vs 10.1%, Cramér’s V = 0.208, *P* < 0.001), patchy infiltration (7.0% vs 5.4%, Cramér’s V = 0.033, *P* < 0.001), pleural effusion (6.6% vs 1.2%, Cramér’s V = 0.144, *P* < 0.001), and atelectasis (5.0% vs 1.7%, Cramér’s V = 0.095, *P* < 0.001) compared to NMPP patients (Table 1).

**TABLE 1:** Demographic of tNGS-positive patients in respiratory samples^*c*^^*,^d^*^

Parameters	*Mycoplasma pneumoniae* infection			Non-*Mycoplasma pneumoniae* (*n* = 5,657)	*P*-value^*a*^	*P*-value^*b*^
	Total (*n* = 4,397)	Solely (*n* = 1,515)	Co-infection (*n* = 2,882)			
Age (years), median (IQR)	6.1 (4.0, 8.1)	7.1 (5.1, 8.8)	5.9 (3.4, 7.7)	2.5 (1.2, 3.9)	**<0.001**	**<0.001**
Male, *n* (%)	2,388 (54.3)	784 (51.8)	1,604 (55.7)	3,278 (58.0)	**0.013**	**<0.001**
Hospital stay duration, median (IQR)	6 (5, 7)	6 (5, 7)	6 (5, 7)	5 (4, 6)	0.072	**<0.001**
Urban population	4,010 (91.2)	1,404 (92.7)	2,606 (90.4)	5,051 (89.3)	**0.012**	**<0.001**
Underlying disease (*n*, %)	57 (1.3)	14 (0.9)	43 (1.5)	92 (1.6)	0.114	0.174
Clinical symptoms (*n*, %)						
Fever	3,801 (86.4)	1,331 (87.9)	2,470 (85.7)	3,749 (66.3)	0.051	**<0.001**
Cough	3,486 (79.3)	1,180 (77.9)	2,306 (80.0)	3,230 (57.1)	0.098	**<0.001**
Wheezing	311 (7.1)	66 (4.4)	245 (8.5)	643 (11.4)	**<0.001**	**<0.001**
Dyspnea	33 (0.8)	7 (0.5)	26 (0.9)	63 (1.1)	0.108	0.063
Vomiting	98 (2.2)	25 (1.7)	73 (2.5)	72 (1.3)	0.059	**<0.001**
Diarrhea	100 (2.3)	21 (1.4)	79 (2.7)	110 (1.9)	**0.004**	0.251
Clinical management (*n*, %)						
ICU admission	67 (1.5)	16 (1.1)	51 (1.8)	209 (3.7)	0.061	**<0.001**
Oxygen therapies	197 (4.5)	59 (3.9)	138 (4.8)	709 (12.5)	0.173	**<0.001**
Ventilatory support	20 (0.5)	5 (0.3)	15 (0.5)	107 (1.9)	0.372	**<0.001**
Corticosteroid use	991 (22.5)	361 (23.8)	630 (21.9)	639 (11.3)	0.138	**<0.001**
Imaging features (*n*, %)						
Consolidation	1,136 (25.8)	409 (27.0)	727 (25.2)	569 (10.1)	0.202	**<0.001**
Patchy infiltration	307 (7.0)	105 (6.9)	202 (7.0)	303 (5.4)	0.923	**<0.001**
Pleural effusion	291 (6.6)	123 (8.1)	168 (5.8)	69 (1.2)	**0.004**	**<0.001**
Atelectasis	221 (5.0)	87 (5.7)	134 (4.6)	95 (1.7)	0.115	**<0.001**
Macrolide-resistance mutation in the 23S rRNA, *n* (%)						
A2063G	3,449 (78.4)	1,230 (81.2)	2,219 (77.0)	*–*	**<0.001**	*–*
A2064G	11 (0.25)	2 (0.13)	9 (0.31)	*–*	0.35	*–*
A2067G	2 (0.05)	1 (0.07)	1 (0.03)	*–*	1	*–*
A2063G + A2064G	1 (0.02)	0 (0)	1 (0.03)	*–*	1	*–*
Total	3,463 (78.8)	1,233 (81.4)	2,230 (77.4)	*–*	**<0.001**	*–*

^*a*^ *P*-value, between patients solely infected with *Mycoplasma pneumoniae* and co-infected with *Mycoplasma pneumoniae* and other pathogens.

^*b*^ *P*-value, between patients with *Mycoplasma pneumoniae* infection and non-*Mycoplasma pneumoniae* infection.

^*c*^ Hospital stay duration and clinical management data were analyzed using multivariate regression models to adjust for potential confounding factors, while Chi-square tests were employed for group comparisons of other categorical variables. In cases where the expected frequencies in any cell were below 5, Fisher's exact test was applied instead of the Chi-square test.

^*d*^ “–” indicates not applicable; Bold values denote statistically significant differences (p < 0.05).

### Age, gender distribution, and hospitalization

In an analysis of tNGS-positive respiratory samples from 10,054 children with pneumonia, the associated age and sex distribution was assessed. The median age of MP-positive patients was 6.1 years, significantly higher than the median age of 2.5 years of the MP-uninfected patients (*r* = 0.48, *P* < 0.001). When stratified by type of infection in the MP-positive group, the median age of children infected with MP only was 7.1 years, which was significantly higher than that of children with co-infections, which was 5.9 years (*r* = 0.14, *P* < 0.001). There was also a statistically significant difference in gender distribution, with a higher proportion of males in the NMPP group (58.0%) compared to the MP-positive group (54.3%) (Cramér’s V = 0.036, *P* < 0.001). Of note, the proportion of men in the MP-positive group was higher for co-infections (55.7%) than for mono-infections (51.8%, Cramér’s V = 0.038, *P* = 0.013). In addition, the median hospital stay in children with MPP was 6 days (IQR: 5–7), compared to 5 days (IQR: 4–6) in children with NMPP, representing a significant difference between the two groups (*r* = 0.78, *P* < 0.001). However, within the MPP group, no significant difference in hospital length of stay was found between patients with sole-MPP and co-infections MPP (Table 1).

### Prevalence and characteristics of macrolide-resistant *M. pneumoniae*

In this study, we investigated the prevalence of macrolide-resistant mutations in MP among 4,397 pediatric patients with confirmed MP infection, focusing on age-dependent distribution, mutation types, and sample source variation. Macrolide-resistant cases were prevalent in all age groups. However, the proportion of macrolide-resistant cases showed significant differences between age groups (Cramér’s V = 0.23, *P* < 0.001). In particular, children aged >11 years had a significantly higher proportion of macrolide-resistant cases than children aged <1 year (88.5% vs 75.6%, Cramér’s V = 0.161, *P* = 0.0013) and children aged 6–11 years (88.5% vs 76.6%, Cramér’s V = 0.075, *P* < 0.001). In addition, children with macrolide-resistant infections had a significantly longer hospital stay than children with non-mutant infections (6 days vs 5 days, *r* = 0.77, *P* < 0.001). No significant difference in mutation rates was found between genders (Table 2).

**TABLE 2:** Differences in the clinical characteristics of participants stratified by macrolide-resistance mutation status^*a*^

Variable	Total (*n* = 4,397)	Mutant (*n* = 3,463)	Non-mutant (*n* = 934)	*P*-value^*b*^
Ages				**<0.001**
<1 year old, *n* (%)	205 (4.7)	155 (75.6)	50 (24.4)	
1–3 years old, *n* (%)	500 (11.4)	409 (81.8)	91 (18.2)	
3–6 years old, *n* (%)	1,253 (28.5)	1,007 (80.4)	246 (19.6)	
6–11 years old, *n* (%)	2,247 (51.1)	1,722 (76.6)	525 (23.4)	
>11 years old, *n* (%)	192 (4.4)	170 (88.5)	22 (11.5)	
Male, *n* (%)	2,388 (54.3)	1,878 (54.0)	510 (55.3)	0.491
Hospital stay duration, median (IQR)	6 (5, 7)	6 (5, 7)	5 (4, 6)	**<0.001**

^*a*^ The overall differences in macrolide resistance between age groups were assessed using the Chi-square test. Pairwise comparisons revealed significant differences, with the >11 year group showing a higher proportion of macrolide resistance compared to the <1 year and 6–11 year groups (*P* = 0.013 and *P* = 0.004, respectively). Hospital stay duration data were analyzed using multivariate regression models to adjust for potential confounding factors.

^*b*^ Bold values denote statistically significant differences (p < 0.05).

The macrolide resistance rate among MP isolates was 78.8% (3,463/4,397). The A2063G mutation in domain V of the 23S rRNA gene proved to be the dominant mutation, which was detected in 78.4% of the samples analyzed. This mutation was significantly more frequent in the samples with sole-MPP (81.2%) than in those with co-infections (77.0%, Cramér’s V = 0.051, *P* < 0.001). Other mutations, including A2064G and A2067G, were less frequent, and their distribution did not vary according to the number of pathogens (Table 1). Of note, the prevalence of macrolide-resistance mutations was significantly lower in BALF (66.3%) than in sputum (78.3%) and throat swabs (79.9%) (Cramér’s V = 0.100 and 0.130, *P* < 0.001) (Table 3).

**TABLE 3:** Positivity rates of tNGS and *Mycoplasma pneumoniae*, microbiome profile, alongside occurrences of resistance mutations in pediatric pneumonia cases, across various specimen types^*a*^

Parameters	Specimen type			*P*-value^*b*^
	BALF (*n* = 447)	Throat swab (*n* = 7,943)	Sputum (*n* = 1,833)	
Positive for tNGS	440 (98.4)	7,799 (98.2)	1,815 (99.0)	0.052
Positive for *Mycoplasma pneumoniae*	389 (87.0)	3,386 (42.6)	622 (33.9)	**<0.001**
Solely	162 (36.2)	1,235 (15.6)	118 (6.4)	**<0.001**
Co-infection	227 (50.8)	2,151 (27.1)	504 (27.5）	**<0.001**
Microbiome profile				
RV	71 (15.9)	603 (7.6)	130 (7.1)	**<0.001**
EBV	60 (13.4)	165 (2.1)	78 (4.3)	**<0.001**
*H. influenzae*	38 (8.5)	643 (8.1)	165 (9.0)	0.44
*S. pneumoniae*	35 (7.8)	546 (6.9)	166 (9.1)	**0.005**
PIV	35 (7.8)	160 (2.0)	37 (2.0)	**<0.001**
RSV	28 (6.3)	137 (1.7)	27 (1.5)	**<0.001**
*C. albicans*	15 (3.4)	41 (0.5)	67 (3.7)	**<0.001**
*hCoV*	14 (3.1)	79 (1.0)	20 (1.1)	**0.002**
CMV	12 (2.7)	116 (1.5)	31 (1.7)	0.11
hMPV	11 (2.5)	172 (2.2)	34 (1.9)	0.622
HBoV	10 (2.2)	72 (0.9)	19 (1.0)	0.054
CV	9 (2.0)	97 (1.2)	20 (1.1)	0.28
AdV	9 (2.0)	102 (1.3)	13 (0.7)	**0.037**
HHV	8 (1.8)	40 (0.5)	50 (2.7)	**<0.001**
*S. aureus*	7 (1.6)	85 (1.1)	57 (3.1)	**<0.001**
*Bordetella pertussis*	7 (1.6)	30 (0.4)	8 (0.4)	**0.014**
HSV	5 (1.1)	35 (0.4)	7 (0.4)	0.188
*Moraxella catarrhalis*	4 (0.9)	52 (0.7)	24 (1.3)	**0.026**
*Klebsiella pneumoniae*	3 (0.7)	83 (1.0)	19 (1.0)	0.718
*A. baumannii*	2 (0.5)	165 (2.1)	35 (1.9)	0.054
EV	2 (0.5)	95 (1.2)	24 (1.3)	0.312
*Candida glabrata*	2 (0.5)	19 (0.2)	52 (2.8)	**<0.001**
Macrolide-resistance mutation in the 23S rRNA, *n* (%)				
A2063G	258 (66.3)	2,704 (79.9)	487 (78.3)	**<0.001**
A2064G	1 (0.3)	8 (0.1)	2 (0.1)	0.636
A2067G	0 (0)	2 (0.03)	0 (0)	0.604
A2063G + A2064G	1 (0.3)	0 (0)	0 (0)	–

^*a*^ The statistical significance between the three groups was analyzed using the Chi-square test. In cases where the expected frequencies in any cell were below 5, Fisher’s exact test was applied instead of the Chi-square test. Pairwise comparisons were performed using the Benjamini-Hochberg correction to adjust for multiple comparisons.

^*b*^ “–” indicates not applicable; Bold values denote statistically significant differences (p < 0.05).

### tNGS reveals a high prevalence of MP and a diverse microbiome in respiratory samples of pediatric patients

tNGS elucidated the pathogen landscape in a pediatric cohort with lower respiratory tract infections and showed a remarkably high pathogen detection rate. In particular, BALF showed a positivity rate of 98.4%, closely followed by throat swabs with 98.2% and sputum samples with 99.0%. Among the pathogens detected, MP was identified as the predominant microorganism in BALF, occurring in both single infections and co-infections with positivity rates of 36.2% and 50.8%, respectively, significantly outperforming throat swabs and sputum samples (Cramér’s V = 0.118–0.359, *P* < 0.001).

Microbiome profiling revealed the complex microbial environment associated with MP positivity. In particular, *Rhinovirus* and *Epstein-Barr virus* were significantly represented in BALF samples, with positivity rates of 15.9% and 13.4%, respectively. In addition, a higher incidence of *parainfluenza virus*, *respiratory syncytial virus*, *human coronavirus*, *adenovirus*, and *Bordetella pertussis* was found in BALF compared to throat swabs and sputum. Analysis of the sputum revealed that *Streptococcus pneumoniae* (9.1%), *Haemophilus influenzae* (9.0%), and *Rhinovirus* (7.1%) were the main pathogens, a pattern that was mirrored in the throat swabs, with *Haemophilus influenzae* (8.1%), *Rhinovirus* (7.6%), and *Streptococcus pneumoniae* (6.9%) at the top of the detection list. The analysis also revealed increased detection rates for S*treptococcus pneumoniae, Human herpesvirus*, *Staphylococcus aureus*, *Moraxella catarrhalis*, *Candida albicans*, and *Candida glabrata* in sputum compared to other sample types (Table 3).

### Inflammatory markers and lymphocyte subpopulations

**Fig 4:**
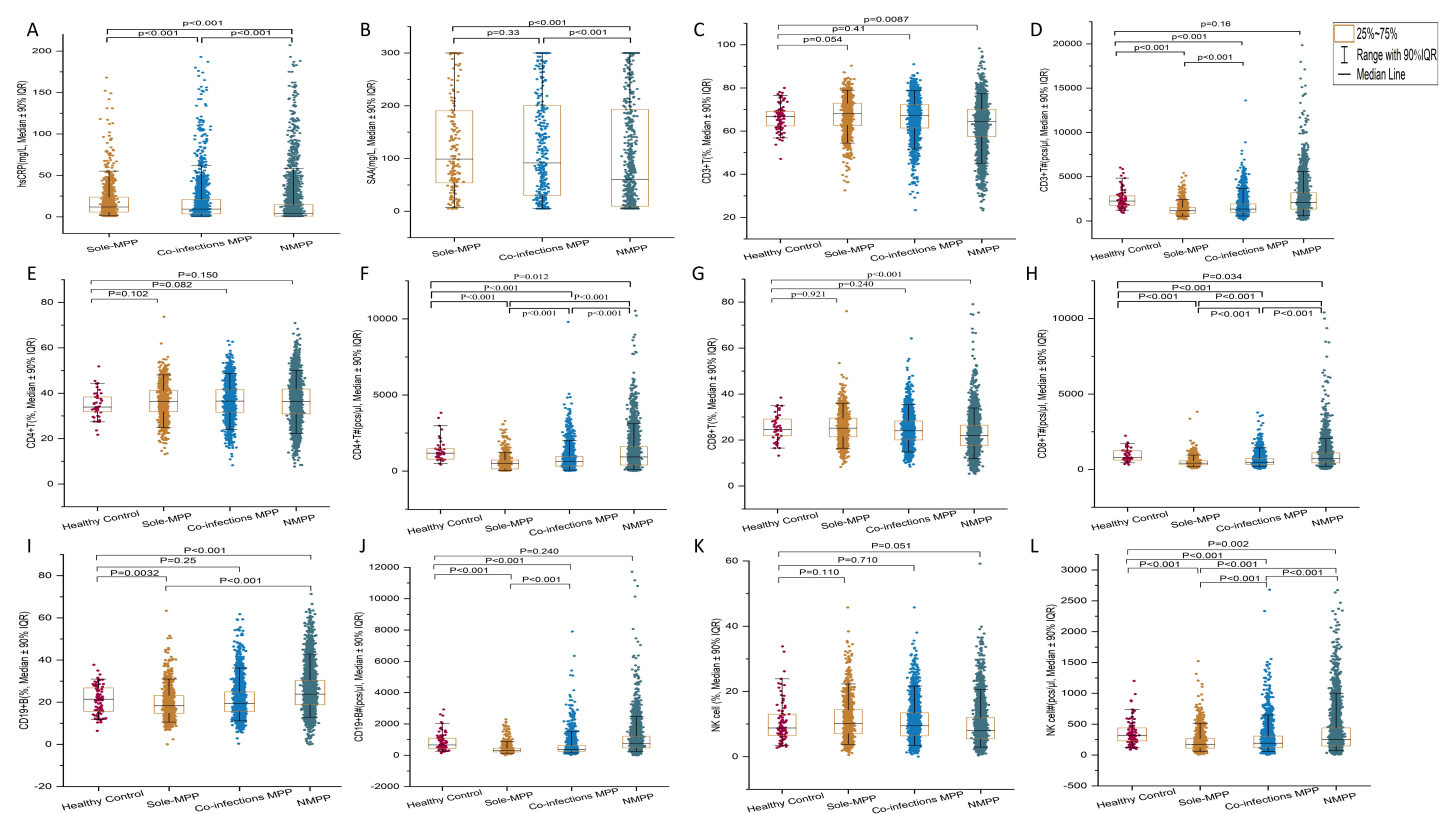
(A–L) Distribution of inflammatory markers and lymphocyte subpopulations in pediatric pneumonia. Scatter plots with box lines overlaid with individual data points representing the levels of hsCRP (**A**), SAA (**B**), the percentages and absolute counts of CD3^+^ T cells (**C and D**), CD4^+^ T cells (**E and F**), CD8^+^ T cells (**G and H**), CD19^+^ B cells (**I and J**), and NK cells (**K and L**) in the peripheral blood of the groups. To compare the groups, we used the Mann-Whitney *U* test for pairwise comparisons between the groups. The results were adjusted for multiple comparisons using the Benjamini-Hochberg correction.

In our pneumonia study, all patient groups (sole-MPP, co-infections MPP, and NMPP) had elevated hsCRP and SAA levels outside the normal range, with significant group differences (*r* = 0.404–0.789, *P* < 0.001). In particular, the sole-MPP group had the highest median hsCRP value of 11.8 mg/L (IQR: 5.6–23.9), while the MPP group with co-infections had 9.4 mg/L (IQR: 3.5–21.2), and the NMPP group had the lowest value of 3.9 mg/L (IQR: 0.85–14.8) (Fig. 4A). SAA levels were significantly higher in both the sole-MPP group, with a median value of 99.0 mg/L (IQR: 52.8–190.7), and the MPP group with co-infections, with 91.5 mg/L (IQR: 30.2–200.6), than in the NMPP group, which had a median value of 60.7 mg/L (IQR: 9.7–193.1) (*r* = 0.125–0.133, *P* < 0.001). Nevertheless, there was no significant difference in SAA values between the sole-MPP and co-infections MPP groups (*P* = 0.33) (Fig. 4B).

In our study, we found no significant differences in the percentages of CD3^+^ T cells, CD4^+^ T cells, CD8^+^ T cells, and NK cells between pediatric patients with MP infections (both sole and co-infections) and healthy controls (*P* = 0.054–0.921). The median values for these markers were comparable in all groups. When analyzing the absolute counts, both sole-MPP and co-infection MPP groups showed a significant reduction in CD3^+^ T cells, CD4^+^ T cells, CD8^+^ T cells, and CD19^+^ B cells compared to healthy controls (*r* = 0.136–0.342, *P* < 0.001). For CD3^+^ T cells, the median counts were 1,146 cells/µL (IQR: 858–1,129) in sole-MPP and 1,343 cells/µL (IQR: 970.3–1,922) in co-infections MPP, compared to 2,240 cells/µL (IQR: 1,332.5–3,181) in controls. CD4^+^ T cells had median counts of 494 cells/µL (IQR: 138–726) in sole-MPP, 628 cells/µL (IQR: 324.8–965.8) in co-infections MPP, and 1,180 cells/µL (IQR: 837–1,495) in controls. Similarly, CD8^+^ T cells were reduced with median counts of 420 cells/µL (IQR: 313–582) in sole-MPP, 482 cells/µL (IQR: 338–718) in co-infections MPP, and 758 cells/µL (IQR: 600–1,104) in controls. For CD19^+^ B cells, the median value was 301 cells/µL (IQR: 214–450) in the sole-MPP and 375 cells/µL (IQR: 254–635.5) in the co-infections MPP group, compared to 657 cells/µL (IQR: 472–1,222) in controls. The sole-MPP group had a lower percentage of CD19^+^ B cells compared to both the NMPP group and the healthy control group. The median value was 18.3% (IQR: 14.8%–23.2%) and thus differed significantly from the 23.8% (IQR: 18.8%–30.4%) of the NMPP group and the 21.4% (IQR: 15.7%–26.8%) of the healthy control group (*r* = 0.295 and 0.211, *P* < 0.001 and *P* = 0.0032). The study also found the absolute count of NK cells was significantly lower in both MP infection groups compared to the NMPP group and healthy controls (*r* = 0.156–0.245, *P* < 0.001). The sole-MPP group had a median value of 174 cells/µL (IQR: 111–267) and the MPP co-infections group had 192 cells/µL (IQR: 120–306), which was significantly lower than the 251 cells/µL (IQR: 146–439.5) of the NMPP group and the 319 cells/µL (IQR: 229–439) of the healthy control group (Fig. 4C through L).

## DISCUSSION

In Wuhan, tNGS has become an established tool for the detection of respiratory tract infections, providing clinicians with a broad and detailed understanding of respiratory tract infection pathogens that exceed the capabilities of conventional diagnostic methods. Importantly, this study demonstrates the direct clinical utility of tNGS, which enables timely and accurate treatment by combining pathogen detection with a patient’s clinical symptoms and imaging studies. This synergy provides for more personalized and effective interventions, particularly in pediatric patients, who may present with complex or atypical respiratory disease. In addition, the large sample size of the study and the inclusion of a special population—children during an MP epidemic—provide robust data that improve the understanding of the epidemiological characteristics of pathogens in the Wuhan region and add significant value to the results. Our study shows a high detection rate (98.4%) of respiratory pathogens by tNGS, which is similar to or slightly higher than other studies, such as Sun et al. (30) (92.8% in BALF samples) and Ma et al. (31) (over 90% in sputum samples from lower respiratory tract infections). The higher rate in our cohort may be due to the comprehensive pathogen panel and rigorous specimen processing, highlighting tNGS as a reliable diagnostic tool for respiratory tract infections. In the past, the positivity rate of MP was between 16% and 40%. Rivaya’s study (32,33) (16.8% in children with pneumonia) and the 2019–2020 Taiwanese study (39.4% in children) show similar trends (33). Our study, based on previous research by Xu et al. (34), observed a slight increase in MP positivity rate from 40.6% (511/1,259) to 43.0% (4,397/10,223) from October 2022 to October 2023, with a marked increase from March 2023, peaking in October at 63.17%. Consistent with previous findings (33), our study showed that fever (86.4%) and cough (79.3%) were the predominant clinical symptoms in MPP patients. Similar to previous reports (35, 36), imaging features, such as lung consolidation and pleural effusion, are more likely to occur in MPP compared with other types of pneumonia. In our study, corticosteroid use was documented in 22.5% of MPP patients. In particular, lung consolidation observed on imaging may serve as an indicator for high-dose corticosteroid therapy, highlighting the importance of tailoring treatment strategies based on imaging findings. While ICU admission, ventilatory support, patchy infiltration, and atelectasis showed statistically significant differences between NMPP and MPP patients, the effect sizes for these differences were small. This suggests that their clinical relevance may be limited despite statistical significance, emphasizing the need to carefully interpret the results in the context of patient management.

MP infections can occur throughout the year but are more common in the autumn and winter in northern China and in the summer and autumn in southern China, with an epidemic cycle every 3–7 years (37–39). China experienced peaks in 2012, 2016, and 2019 (40, 41). In 2023, MP infections in northern China followed the usual seasonal and cyclical pattern, peaking in autumn and winter, while southern China experienced a major outbreak in summer and autumn 2019 (37). As a city in central southern China, Wuhan’s seasonal prevalence fits these patterns. Furthermore, this resurgence can be attributed to an “immunity gap” and “immunity debt” following the COVID-19 pandemic, suggesting a greater prevalence of MP infections due to reduced immune defenses in children against common respiratory pathogens (42, 43). These observations underscore the urgent need for broader surveillance, including multicenter monitoring, and comprehensive research to fully understand the trends of MP infections and develop effective prevention and treatment strategies.

Our study shows that the primary form of MP infection in pediatric pneumonia is co-infection with MP and other pathogens, accounting for 65.6% of cases. The most frequently identified co-infecting pathogens were *Haemophilus influenzae, rhinovirus, Streptococcus pneumoniae,* and *Epstein-Barr virus*. This is in contrast to the findings of Sung et al. (44) in which only 45.4% of patients with MPP had co-infections, with *rhinovirus* being the most common co-infecting pathogen. The variance in the prevalence of MPP with co-infection could be due to regional differences or different detection methods. There are reports indicating that members of the Herpesviridae family, such as *Epstein-Barr virus* and *cytomegalovirus*, can be reactivated in ICU patients (45). Such reactivation is associated with increased morbidity and mortality, suggesting that disturbances in the regulation of the immune system may play a role in the activation of these viruses, along with the clinical presentation of the child (symptoms such as fever, sore throat, swollen lymph nodes, and loss of appetite), viral serology, and the presence of immunosuppressive diseases (such as leukemia or lymphoma). The distinction between primary infection and the reactivation of viruses such as *Epstein-Barr virus* is crucial for the choice of an appropriate clinical treatment strategy.

In our study, the primary pattern of infection in pediatric cases of non-MP pneumonia was bacterial and viral co-infections, with *Haemophilus influenzae, respiratory syncytial virus,* and *Streptococcus pneumoniae* predominating. In particular, *Haemophilus influenzae* and *Streptococcus pneumoniae* were highly detected in both MP and non*-*MP pneumonia cases, possibly due to the fact that their vaccines are not part of the Chinese mandatory vaccination schedule, resulting in low vaccination coverage. In addition, although *respiratory syncytial virus* was predominant in non-MP infections, its lower prevalence in MP cases warrants further investigation.

Our study sheds light on significant trends in age and gender distribution, as well as the impact of length of hospitalization. Although we observed statistically significant differences in gender distribution between the MP-positive and NMPP groups (e.g., 54.3% vs 58.0%), the small effect size suggests that gender may have a small impact, but it is unlikely to be an important determinant of clinical outcomes or susceptibility to infection. The higher median age of MP-positive individuals compared to uninfected individuals suggests age-related susceptibility or different levels of exposure. The age distribution also distinguishes between sole and co-infections, indicating age-specific immune responses or exposures. Our results show that the median hospitalization for MPP patients is 6 days, which is consistent with the observations of Guo et al. (46) and highlights the significant burden on the healthcare system and the need for effective treatment strategies to relieve the burden on healthcare systems.

Our study shows significant differences in macrolide resistance rates of MP between China, especially Wuhan, and other regions. The resistance rate in our cohort was 78.8%, which is far higher than that in the United States (7.5%), Spain (8%), and Denmark (<2%) (32, 47, 48). This finding highlights the regional differences in the prevalence of MRMP and suggests that the overuse of macrolides in China exacerbates resistance. This complicates therapeutic strategies and requires more judicious use of antibiotics to prevent further resistance, especially given the limited secondary treatment options for pediatric patients. Continued research into new macrolide therapies, alternative drugs, and resistance mechanisms is crucial. Given the high prevalence of MRMP in 2023, second-line anti-*Mycoplasma pneumoniae* drugs, such as second-generation tetracyclines (e.g., minocycline and doxycycline) and fluoroquinolones, should be used immediately if macrolides fail within 72 hours, show poor efficacy, or in severe cases (49). Recent studies suggest that midecamycin, which causes less resistance than other macrolides (e.g., erythromycin and azithromycin), could be a promising option for first-line treatment, especially in regions with high resistance such as China (50, 51). In addition, high macrolide resistance caused by 23S rRNA mutations underscores the urgent need for enhanced surveillance, early warning systems, and robust antibiotic stewardship to contain the spread of resistance and ensure sustainable antibiotic use. These measures are critical to improving patient care and combating antimicrobial resistance worldwide.

Our study reveals that macrolide-resistant MP infections are prevalent in all pediatric age groups, with higher rates of resistance in children older than 11 years, likely due to increased macrolide exposure or age-related factors. Prolonged hospitalization of resistant cases underscores the clinical burden of resistance and highlights the need for age-specific strategies. MP resistance arises mainly from point mutations in domain V of the 23S rRNA gene, particularly at positions 2063, 2064, and 2067, with A2063G being the most common mutation (32, 47, 48, 52, 53). We identified a novel double mutation (A2063G and A2064G) and noted rare mutations reported elsewhere, such as C2622T and C2353T (54, 55), reflecting the evolving diversity of resistance mechanisms. The decline in MRMP infections in Japan, attributed to adapted antibiotic use, emphasizes the importance of prudent macrolide use and vigilant surveillance of emerging variants to control the spread of resistance and avoid treatment failures (56). These measures are crucial to mitigate selection pressure and adapt to the dynamic nature of bacterial resistance.

Our results suggest a higher MP detection rate in BALF than in sputum or throat swabs, alongside a lower incidence of macrolide resistance mutations in BALF. This discrepancy may be due to the fact that BALF is taken directly from the lower respiratory tract and thus provides a clearer picture of pulmonary infections and a higher pathogen concentration. The minimal contamination of BALF with the upper respiratory tract or environmental microbes likely increases the specificity in detecting MP. The lower macrolide resistance observed in BALF samples compared to upper respiratory tract samples is consistent with the findings of Wang et al. (57), who reported a significantly lower detection rate of macrolide resistance in BALF samples compared to sputum samples (45.2% vs 53.1%). This discrepancy could be due to a biological advantage for less resistant strains in the lung microenvironment or to the selective pressure created by prior or concurrent topical treatments commonly used in the upper respiratory tract. Such treatments, including nasal saline irrigation, intranasal corticosteroids, and topical antimicrobials, are widely used in Chinese households and could affect the local microbial environment (58, 59). However, our study did not consider the detailed treatment history of patients, limiting the ability to draw definitive conclusions about the impact of these interventions on resistance patterns. Furthermore, the lack of detailed treatment history in our study limits our ability to draw definitive conclusions about the impact of these interventions on resistance patterns.

While the direct link between the host immune response and MPP remains unclear, we hypothesize that inflammatory markers and lymphocyte subpopulation counts could serve as a predictive tool to discriminate between MP and non*-*MP infections. This approach could enable early and individualized adaptation of treatment. Until now, variations in SAA levels in children with MPP have not been thoroughly investigated. This study shows that there is a rapid increase in serum SAA levels in children with MPP compared to children with NMPP. This increase suggests that there is an increase in the expression of various inflammatory cytokines following MP infection, which in turn accelerates the synthesis and secretion of SAA in the liver. SAA may exacerbate the inflammation associated with MP infection through its pro-inflammatory effects, suggesting a close relationship between SAA serum levels and modulation of immune function in children with MPP (60, 61). Previous studies have shown that T cell activation and cell-mediated inflammatory damage, as well as a cytokine-oriented proinflammatory environment in the airways are essential components of the exacerbation of MPP (62).

The innate and adaptive immune responses play a crucial role in the defense against infections by pathogens and disease progression. Previous studies have shown a close correlation between changes in T-cell subsets and the progression of MP infection (9). MP triggers polyclonal lymphocyte activation, which disturbs the balance of T-cell subsets, impairs the immune system, and affects the proliferation and differentiation of other immune cells. In our study, the median absolute counts of CD3^+^ T cells, CD4^+^ T cells, CD8^+^ T cells, CD19^+^ B cells, and NK cells were significantly lower in patients with MPP than in the healthy control group and in patients with NMPP, which is consistent with the results of the study by Li et al. (63) on children with MP infection in Guangzhou, China. This suggests that both humoral and cellular immunity are involved in pathogenesis after MP infection, leading to a significant loss of lymphocytes. However, our results differ from those of Fan et al. (35), who reported significant differences in the percentages of CD3^+^ and CD19^+^ cells between MPP and NMPP groups. In our study, no significant difference in CD3^+^ percentages was observed between these groups, and a difference in CD19^+^ was only observed between the sole MPP group and both the NMPP and healthy control groups. This discrepancy could be due to the fact that previous studies did not differentiate between sole MP infections and co-infections, which could explain the observed differences. Understanding these immune mechanisms in children with MP infection is critical for clinical evaluation and determining treatment strategies that can significantly contribute to reducing complications and mortality. Although statistically significant differences were observed, the distributions of inflammatory markers and lymphocyte subsets showed considerable overlap between the study groups. This overlap limits their utility as stand-alone diagnostic or prognostic tools. Nonetheless, the observed trends may be valuable in certain clinical contexts, providing insights into the pathophysiology of the disease and information for further research. These results emphasize the importance of combining inflammatory markers and lymphocyte subsets with other clinical and laboratory parameters to improve their diagnostic and prognostic potential. While our study focused on inflammatory markers and T-cell subsets, previous research has emphasized the crucial role of immune cell subtypes, including polymorphonuclear cells, monocytes, and lymphocytes, as well as the essential function of macrophages in clearing MP from the lungs of mice (64). Although we did not collect data on these specific immune cell populations in this study, future prospective studies could investigate their role in MP infection and clearance, particularly by analyzing pediatric BALF.

Our study has some limitations that should be mentioned. First and foremost, laboratory evaluations were performed within the first 24 hours of admission, without subsequent follow-up, limiting our ability to capture potential dynamic changes in biomarkers during the course of infection. In addition, the composition of our healthy control group, limited to 96 children who underwent standard health examinations, presents a stark contrast in sample size compared to the experimental cohorts. This discrepancy raises concerns about statistical representativeness and the potential for bias in the comparative analysis. Furthermore, in this control group, our analysis was limited to lymphocyte subpopulations and relied on established reference ranges for hsCRP and SAA levels, as no direct measurements were available. This may not fully reflect the variability within a healthy pediatric population. In addition to these limitations, our study did not collect detailed data on prior or concurrent topical treatments such as nasal irrigation, corticosteroids, or other locally applied therapies. The lack of this information limits our ability to fully interpret the observed resistance patterns between BALF and upper respiratory tract samples. In addition, our study could not distinguish between pathogen colonization and coinfection, as all tNGS samples were from patients with symptomatic pneumonia, making all detected pathogens potentially relevant to their clinical presentation. In addition, the study was conducted at a single institution, which may limit the applicability of the results to a broader base. Finally, participation in the study was limited during the first months of the tNGS rollout in October 2022 due to logistical adjustments, including staff training and the introduction of standard operating procedures for sample collection. While this may have led to minor biases in early data collection, this limitation is mitigated by the substantial increase in cases recorded in subsequent months, providing a robust data set for analyzing pathogen trends. Future studies should consider the inclusion of longitudinal samples to observe temporal variation in biomarkers, expand control groups to improve comparative robustness, and include a broader range of biomarkers for comprehensive analysis. A multicenter design could also provide a more diverse and representative data set and potentially overcome some of the limitations encountered in our current study.

In conclusion, our study underscores the significant prevalence of MP in pediatric CAP and highlights the critical role of tNGS for accurate pathogen detection and resistance profiling. The results emphasize the urgent need for vigilant antibiotic stewardship in the face of high rates of macrolide resistance. In addition, the identification of specific biomarkers, including hsCRP, SAA, and lymphocyte subpopulations, offers the potential to refine MPP diagnostics and individualize therapeutic strategies. This research highlights the complexity of the epidemiology and pathophysiology of MPP and advocates for advanced diagnostics and comprehensive surveillance to improve clinical outcomes in pediatric pneumonia.
